# The Influence of Nonparental Care on Internalizing and Externalizing Behaviors Across Adolescence: An individual Participant Meta-Analysis

**DOI:** 10.1192/j.eurpsy.2024.318

**Published:** 2024-08-27

**Authors:** K. M. Barry

**Affiliations:** ^1^Social Epidemiology, INSERM; ^2^Social Epidemiology, Sorbonne University, Paris, France

## Abstract

**Introduction:**

In Europe, associations between different types of nonparental care and internalizing and externalizing behaviors in children have not been adequately explored (Gialamas, A et al. J Epiemiol Community Health. 2015). Internalizing and externalizing symptoms in childhood can have lifetime repercussions, thus understanding their risk factors and the potentially protective role of family policies is highly relevant.

**Objectives:**

To explore the associations between different types of nonparental care prior to primary school and internalizing and externalizing behaviors across young adolescence.

**Methods:**

Six parent-offspring prospective birth cohort studies across five European countries within the EU Child Cohort Network (EUCCN) were included in the study. A two-stage individual participant data (IPD) meta-analysis on complete cases was performed. Linear regression models (one for each age group: 5-6 years, 7-9 years, 10-13 years) were applied in each cohort separately and then cohort-specific coefficients and standard errors were combined using random-effects (restricted estimate maximum likelihood (REMD) meta-analysis to attain overall effect estimates. Data were then stratified by socioeconomic position and sex.

**Results:**

There were 74 453 parent-offspring dyads to study children’s internalizing difficulties and 72 462 parent-offspring dyads to study children’s externalizing difficulties. Center-based care attendance was associated with lower levels of internalizing difficulties 5-6 years [-1.13 (95%CI:- 2.68, 0.42), p=0.15]; 7-9 years [-1.38 (95%CI:- 2.85, 0.10), p=0.07]; 10-13 years [-1.06 (95%CI:- 1.95, -0.17), p=0.02]. Children who attended other forms of nonparental care appeared to have higher levels of internalizing difficulties: 5-6 years [0.02 (95%CI:- 1.96, 2.01), p=0.98], 7-9 years [0.91 (95%CI:0.23, 1.58), p=0.009]; 10-13 years [0.52 (95%CI:- 0.23, 1.27), p=0.17]. Other forms of nonparental care (not including center-based care) had a positive association with externalizing symptoms : 5-6 years [2.45 (95%CI:0.35, 4.55), p=0.02]: 7-9 years [2.78 (95%CI: 0.60, 4.95), p=0.01];10-13 years [1.93 (95%CI:-0.45, 4.32), p=0.11]. We found some evidence of effect moderation by the child’s sex and socioeconomic position (SEP).

**Conclusions:**

The results suggest that center-based care may protect children from developing internalizing behaviors, but other forms of nonparental care may put children at more risk of developing more internalizing and externalizing behaviors. Also, factors such as sex and SEP may interact with nonparental care in influencing externalizing behaviors.

**Disclosure of Interest:**

None Declared

